# Axl Expression Stratifies Patients with Poor Prognosis after Hepatectomy for Hepatocellular Carcinoma

**DOI:** 10.1371/journal.pone.0154767

**Published:** 2016-05-16

**Authors:** Jian Liu, Kui Wang, Zhenlin Yan, Yong Xia, Jun Li, Lehua Shi, Qifei Zou, Xuying Wan, Binghua Jiao, Hongyang Wang, Mengchao Wu, Yongjie Zhang, Feng Shen

**Affiliations:** 1 Department of Hepatic Surgery, the Eastern Hepatobiliary Surgery Hospital, Second Military Medical University, Shanghai, China; 2 Department of Clinical Database, the Eastern Hepatobiliary Surgery Hospital, Second Military Medical University, Shanghai, China; 3 Department of Biochemistry and Molecular Biology, Second Military Medical University, Shanghai, China; 4 National Scientific Center for Liver Cancer, Shanghai, China; 5 Department of Biliary Surgery, the Eastern Hepatobiliary Surgery Hospital, Second Military Medical University, Shanghai, China; University of North Carolina School of Medicine, UNITED STATES

## Abstract

**Background:**

Axl is a receptor tyrosine kinase which plays an important role in multiple human malignancies.

**Design:**

The Axl expression was examined in several hepatocellular carcinoma(HCC) cell lines, paired tumor and nontumorous samples. Then, we examined cell growth curve, cell apoptosis and cell migration in SMMC-7721 cells over-expressed with Axl or siRNA against Axl, respectively. Finally, the prognostic value of Axl was investigated in a prospective cohort of 246 consecutive HCC patients undergoing curative hepatoectomy.

**Results:**

We found Axl was positive in 22% of examined tumor tissues and all four cell lines. Over-expressing Axl in SMMC-7721 cells accelerated cell growth, cell migration and inhibited cell apoptosis, while knock-down of Axl exerted opposite effect. Axl expression was closely associated with serum AFP, multiple tumors, absence of encapsulation, microvascular invasion, and advanced BCLC or TNM stage. Patients with positive Axl staining had a higher 5-year recurrence rate (92% vs. 71%, *P*<0.001) and a lower 5-year survival rate (9% vs. 48%, *P*<0.001) than those with negative staining. The multivariate analyses showed that Axl expression was an independent factor for both tumor recurrence (HR: 1.725; 95% CI: 1.219–2.441) and survival (1.847; 1.291–2.642).

**Conclusion:**

Axl expression suggests more aggressive tumor invasiveness and predicts worse prognosis for HCC patients undergoing resection.

## Introduction

Hepatocellular carcinoma (HCC) is one of the common malignancies and the fifth cancer-killer worldwide [1]. Hepatectomy remains the first-line treatment for this malignancy, however, the overall outcome is still unsatisfactory due to the high recurrence rates up to 70% at 5 year for overall patients and about 40% for patients at early stage [2,3]. Additionally, the overwhelming majority of patients are not eligible for hepatectomy because of tumor metastasis at diagnosis [4,5]. The molecular mechanism of HCC recurrence and metastasis is largely less understood so far. Our previous study suggested that over-expression of aspartyl (asparaginal) beta-hydroxylase (ASPH) in tumor tissue is closely associated with invasiveness and recurrence of HCC [6]. We further analyzed expression of potential substrates of ASPH which is characterized by containing EGF-like domain, such as growth arrest-specific gene 6 (Gas6), and their related proteins in HCC. We found Axl, the receptor of Gas6, was also over-expressed in HCC tumor tissues [7].

Axl is a member of Tyro-Axl-Mer (TAM) receptor tyrosine kinase (RTK) family, and Gas6 and protein S are common ligands for the members of TAM-RTK family [8]. These family members have two tandem immunoglobulin (Ig)-like loops followed by two fibronectin III-like modules at their extracellular N terminus, and have a tyrosine kinase domain at their C terminus [9]. Normally, Axl is expressed in multiple embryonic tissues, and involved in mesenchymal and neural development [10]. Axl is mainly activated through auto-phosphorylation of intracellular tyrosine kinase domain, thus inducing PI3K/Akt and Wnt pathway [11]. In human malignancies, Axl was originally identified as an oncogene responsible for malignant transformation in chronic myeloid leukemia, in which increasing Axl expression and Axl/Gas6 interaction sufficiently led to tumorigenesis [12]. In breast cancer, Axl expression was an independent prognostic factor [13]. Also, Axl expression conferred tumor resistance to chemotherapy in gastrointestinal stroma tumor and acute myeloid leukemia [14,15].

Axl is largely less studied in HCC so far. Tsou et al. screened the expression of tyrosine kinase in HCC cell lines and two HCC tumor samples and found that Axl was one of the molecules which was over expressed in tumor tissues and poorly differentiated cell lines [7]. Xu et al. found that Axl was up-regulated in YAP1-induced MIHA cell transformation, revealing Axl might be involved in hepatic cells transformation [16]. However, these studies have not pointed out the role in HCC progression. Another study by He et al. indicated that depletion of Axl in mouse liver cancer cell lines could inhibit tumor invasion *in vivo* [17], but whether it is involved in metastasis of human malignancies has not been investigated yet. The exact cellular function and clinical relevance of Axl expression in human HCC are still unknown.

Here we investigated differential expression of Axl in human HCC cell lines, paired HCC tissue and non-tumorous tissue. Then the effects of Axl on cell proliferation and migration were examined by over-expression and depletion of Axl in HCC cell lines and normal liver cells. We also prospectively studied the prognostic value of Axl in HCC tissue, demonstrating a close association between Axl expression and invasive pathological features, and disease outcomes.

## Materials and Methods

### Cell lines, constructs, and human tissues

Human HCC cell lines, SMMC-7721, Hep3B, HepG2, Hu7 and normal liver cell L02 (gifts of Tumor Immunology and Gene Therapy Center, Eastern Hepatobiliary Surgery Hospital, the Second Military Medicial University, 225 Changhai Road, Shanghai 200438, China), were used for Axl function assay. Human wild-type form of Axl (Axl-WT) and siRNA for Axl (Si-Axl) stable transfectants were prepared for cell transfection. The detailed materials and methods including cell culture, RNA analysis, cell growth in vitro, apoptosis, migration and invasion assay was shown in the (S1 Text).

We randomly collected 20 pairs of fresh tumor tissues (TT) and non-tumorous tissues (NTT) from patients undergoing hepatectomy for HCC between November and December 2004 at the Eastern Hepatobiliary Surgery Hospital (EHBH). All patients had HBV background. The mRNA and protein samples were further prepared from these tissues for biochemical assays, which was shown in the (S1 Text).

### Patients

We then analyzed the prospectively collected data of a cohort of consecutive patients who underwent partial hepatectomy for HCC between January 2004 and February 2005 at the Eastern Hepatobiliary Surgery Hospital (EHBH). The inclusion criteria were patients with: (1) no history of previous anti-cancer therapy; (2) no history of other malignancies; (3) Child-pugh A class of liver function; (4) no distant tumor metastasis;(5) completed resection of macroscopic liver tumors confirmed by intra-operative ultrasound (IOUS); and (6) pathologically proven HCC.

Histopathological study of the resected specimens was carried out independently by three pathologists who came to a consensus by discussion if there was any controversy. HCC was histologically classified using Edmondson-Steiner classification [18] and clinically classified using BCLC staging [19] and TNM staging systems(6^th^ edition) [20].

The microarray and immunohistochemistry and clinical data from included patients were collected for the prognostic study. The construction of tissue microarray and evaluation of immunohistochemistry was presented in (S2 Text).

The study was approved by the Institutional Ethics Committee of the Eastern Hepatobiliary Surgery Hospital. Written informed consent was obtained from all patients.

### Follow-up

Patients were followed-up every 2 month in the first 2 years after surgery, and every 3 to 6 month thereafter. At each of the follow-up visits, a detailed physical examination, serum AFP, CA19-9, CEA, liver function test, and abdominal ultrasound was carried out. A contrast-enhanced CT or MRI was performed every 6 months or earlier when tumor recurrence or metastases was suspected. Further investigation was carried out when clinically indicated. Tumor recurrence was defined as the appearance of a newly detected tumor confirmed on two radiological imaging with or without elevation of serum tumor markers. The treatment modalities after relapse were described in our previous study [6].

### Statistical analysis

Overall survival (OS) and time to recurrence (TTR) were used as primary endpoints. OS was defined as the interval between hepatectomy and death or the date of last follow-up. TTR was calculated from surgery to the date when recurrence was diagnosed.

All statistical calculations were carried out using the SPSS.13 software. The χ2 test or Fisher’s exact test were used to compare qualitative variables, while continuous variables were compared using Student’s t-test or Mann-Whitney test for variables with an abnormal distribution. Receiver operating characteristic curve analysis was used to determine the optimal cut-offs of continuous variables. Survival curves were calculated by the Kaplan-Meier method and compared using the log-rank test. The Cox proportional hazards model was used to determine the independent factors for tumor recurrence and, patient’s survival based on the variables selected by univariate analysis. Differences were considered statically significant if p<0.05.

## Results

### AXL expression in HCC samples and cell lines

**Fig 1:**
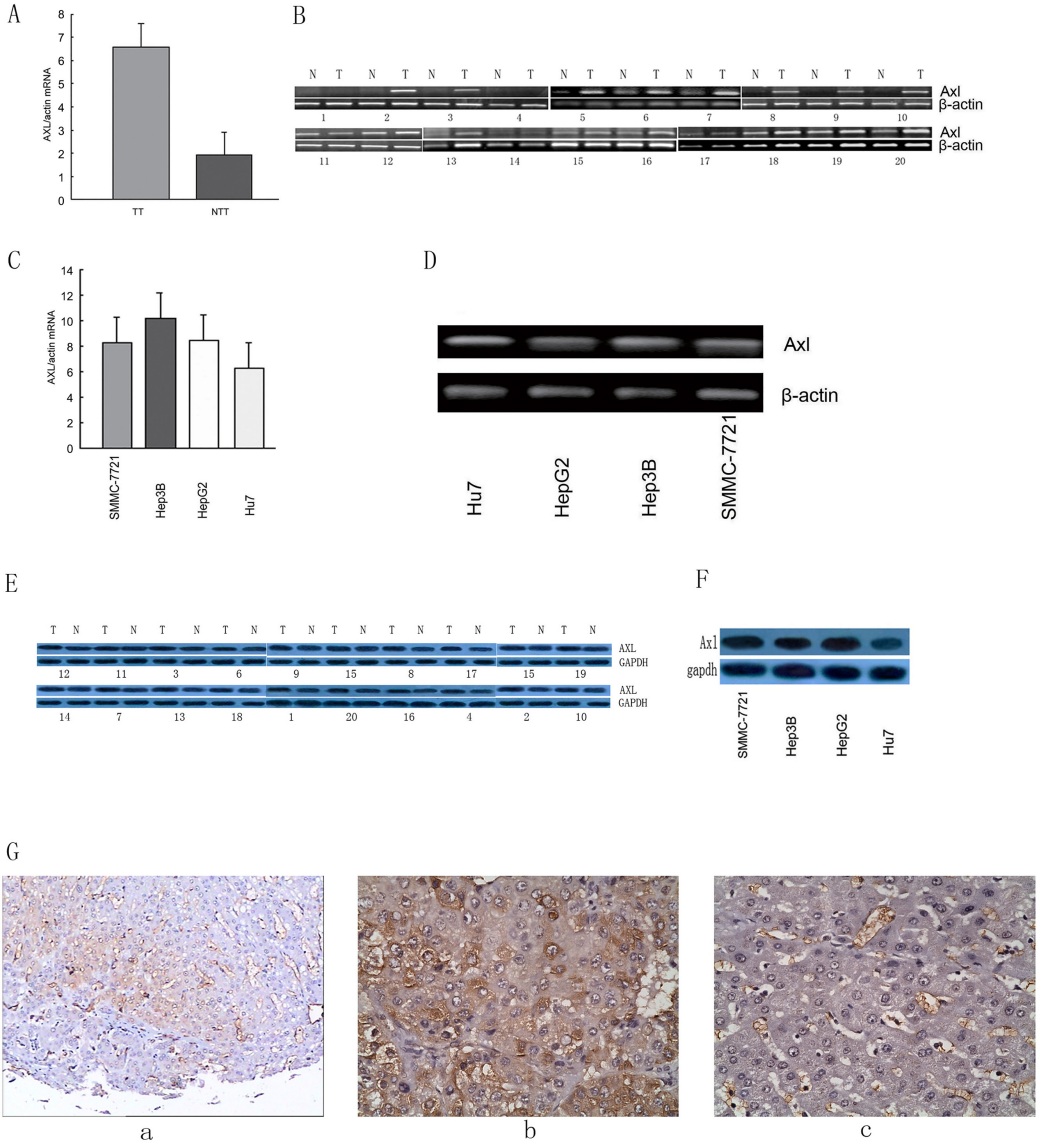
Expression of AXL in HCC specimens and cell lines. Results of examining Axl expression in tumor tissues and paired non-tumorous tissues by real-time RT-PCR (A), RT-PCR (B) and western blot (E), respectively. Results of examining Axl expression in SMMC-7721, Hep3B, HepG2, Hu7 cell lines by real-time RT-PCR (C), RT-PCR (D) and western blot (F), respectively. β-actin was used as internal control. G: Representative images of immunohistochemical staining of AXL in paired tumor (*b*) and non-tumorous tissues (*c*). a: the lower magnification field. Bars, ±SD.

We firstly assayed and compared Axl mRNA levels in 20 TTs and their matched NTTs by real-time RT-PCR and RT-PCR (Fig 1A and 1B). Axl mRNA was over expressed in all TTs as compared to paired NTTs. Also, HCC cell lines of SMMC-7721, HepG2, Hep3B, and Hu7 showed a high mRNA level of Axl (Fig 1C and 1D). Axl proteins were further examined in paired TT and NTT and four HCC cell lines and found highly expressed in tumor tissues and HCC cell lines (Fig 1E, and S1 and S2 Figs). The immunostaining results showed intensive Axl expression throughout the tumor specimen while negative staining on the liver parenchyma cells in the corresponding non-tumorous tissue (Fig 1Fa, 1Fb and 1Fc). Collectively, Axl was over expressed in HCC.

### Expression of Axl affects HCC cell growth and invasiveness in vitro

**Fig 2:**
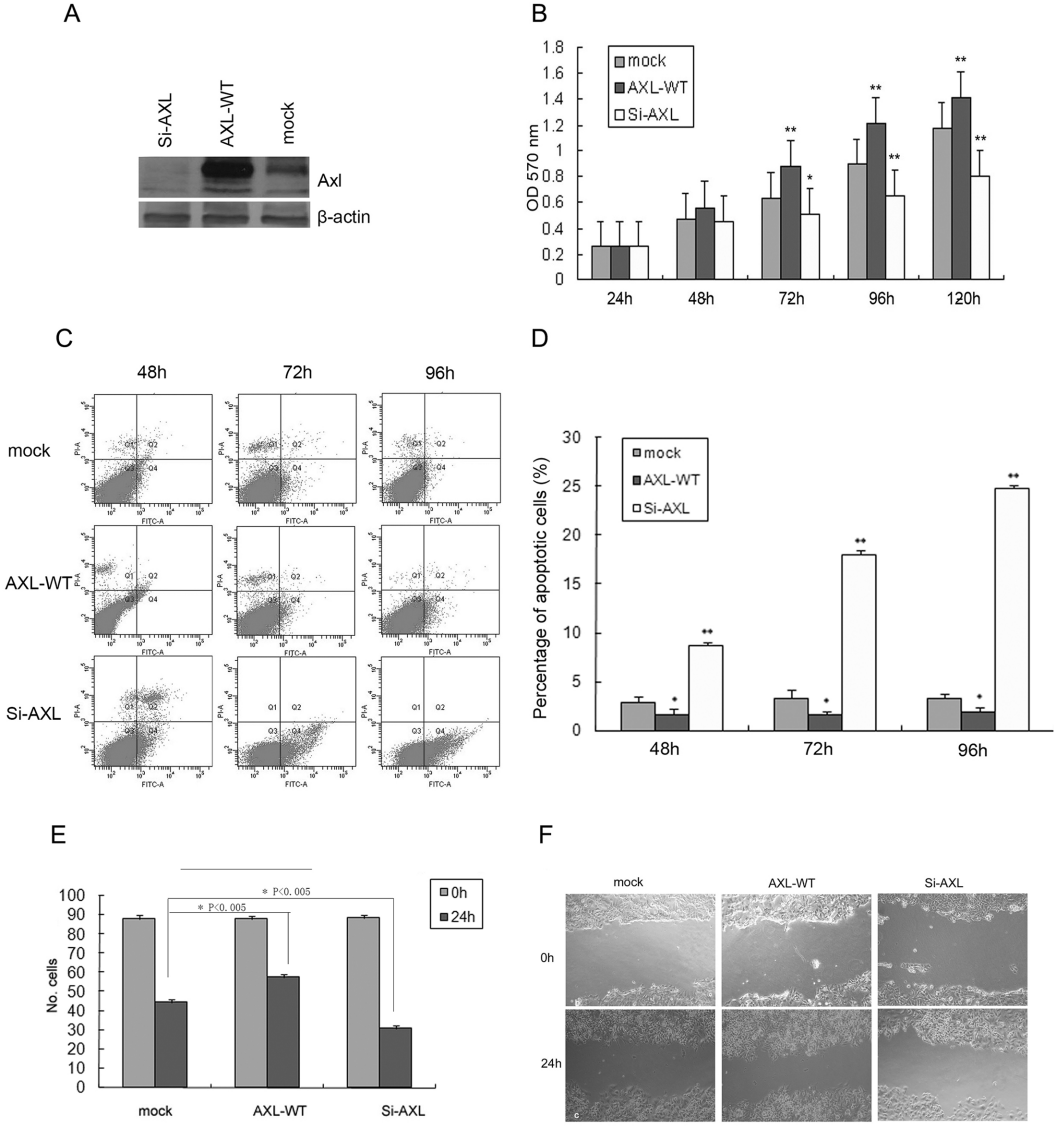
Effects of AXL expression on cell growth and invasiveness of SMMC-7721. A: Western blot analysis of cell lysate expressing the control vector (mock), the wild-type AXL (AXL-WT, stable transfection), and RNAi AXL (Si-AXL, transient transfection after 72 hours). B: Cell growth curve of cells transfected with constructs as indicated by MTT assay. C, D: Representative plots of flow cytometry and statistical results of cell apoptosis of cells transfected by constructs as indicated by propidium iodide and annexin V-FITC staining. Bars, ±SD. *, *P*<0.05 versus mock, **, *P*<0.001 versus mock. E: Statistical results of cells transfected with constructs as indicated in transwell assay. *, *P*<0.05 versus mock, **, *P*<0.001 versus mock. F: Representative image of cells transfected with constructs as indicated in the wound healing assay. Mock: control vector; AXL-WT: the wild-type AXL; si-Axl: transient transfection after 72 hours.

As shown in Fig 2A, we over-expressed and depleted Axl in SMMC-7721 cells through transfection of wild-type of Axl (Axl-WT) or siRNA against Axl (Si-Axl), respectively. Axl over-expression changed cells morphology, displaying a diffusive and invasive feature. However, some cells transfected with si-Axl became detached and exhibited a round shape, while cells in control group grew in a tightly monolayer.

As shown in Fig 2B and S3 Fig, over-expression or depletion of Axl in HCC cells greatly promoted or inhibited cell growth. Cell growth rate of Axl-depleted cells was significantly inhibited as compared to the mock and Axl over-expressed cells 48 hours after transfection. The inhibition effect was more distinct at 72 hours and thereafter. In addition, cells with Axl over-expression showed an accelerated growth rate as compared to other two groups 48 hours after transfection. The similar results were also obtained in other HCC cell lines, even in normal liver cell(L02). But the inhibition effect was more remarkable in HCC cell lines than that of L02(S3 Fig). The accelerated growth rate was faster in L02 than in HCC cell line. The most probable reason for this phenomenon was the lower expression of Axl in L02 than that in HCC cell lines.

It has been reported that activation of Axl receptor could induce cell cycle re-entry in serum-starved NIH 3T3 cells and thereby enhance cell survival [21]. We then examined whether Axl expression affected the apoptosis of HCC cells and L02. Through analysis of cell apoptosis by flow cytometry, we found that depletion of Axl in HCC cells greatly promoted cell apoptosis, as evidenced by a higher apoptotic rates in si-Axl transfection group than those in the control group (8.4%, 18.0%, and 24.4% vs. 2.3%, 4.0%, and 3.2% at 48, 72 and 96 hours, respectively, *P*<0.001 for all, Fig 2C and 2D). As shown in S4 Fig, the apoptotic rates were 15.56% v 2.06% for Hep3B, 13.39% v 1.13% for HepG2, 11.54% v 3.09% for Hu7, and 3.495 v 2.07% for L02 72 hours after transfection respectively(P<0.001 for all). In contrast, cells with over-expressed Axl had lower apoptotic rates than those in the control group at all time points (1.2%, 1.8% and 2.3% vs. 2.3%, 4.0%, and 3.2%, respectively, *P*<0.05 for all, Fig 2C and 2D). The apoptotic rates were 1.43% v 2.06% for Hep3B, 0.98% v 1.13% for HepG2, 0.98% v 3.09% for Hu7, and 1.43% v 2.07% for L02 72 hours after transfection respectively(P<0.05 for all, S4 Fig).

The role of Axl in invasiveness of HCC cells was tested by transwell and wound healing assay. After cell migration in the transwell for 24 hours, more cells over-expressed with WT-Axl migrated to the bottom chamber than those in the mock group did, while the number of migrated cells in the Axl-silenced group was the least (Fig 2E, and S5 Fig). In the wound healing assay, Axl over-expressed cells also showed the fastest wound recovery, while the mock cells ranked second and the Axl-silenced cells showed slowest wound recovery (Fig 2F, and S6 Fig). The similar results were also obtained in Hep3B, HepG2, Hu7. Even in the normal liver cell line L02, over expression of Axl promoted liver cell migration, while depleting Axl inhibited. The results were shown in supplementary materials.

### Axl expression is correlated with invasive clinicopathological characteristics

During the study period, 246 consecutive patients meeting the inclusion criteria were enrolled and underwent clinical analysis, of which 222(222/246, 90.2%) had HBV background. Of them, 54 had Axl expression in TTs as compared with NTTs. In their samples, positive staining of Axl was absent in the non-tumorous liver parenchyma, but observed in the microvascular cells.

Correlation regression analysis indicated that intratumoral Axl expression was significantly associated with some critical pathological characteristics of tumor invasiveness including multiple tumor numbers (*P*<0.001), absence of tumor capsule (*P* = 0.003), microvascular invasion (MVI, *P* = 0.004), preoperative positivity of serum AFP (*P* = 0.003), advanced BCLC stage (*P*<0.001) and TNM stage (*P* <0.001) (Table 1).

**Table 1:** Correlation between intratumoral AXL expression and clinicopathologic features. **Abbreviations**: AFP, alpha-fetoprotein; HBsAg, hepatitis B surface antigen; HBeAg, hepatitis E antigen; PLT, platelet count; TBIL total bilirubin; ALB, albumin; ALT, alanine aminotransferase; PVTT, portal vein tumor thrumbus; MVI, microvascular invasion.

Variable	AXL expression (n = 246)		
	Negative (n = 192)	Positive (n = 54)	*P*
Age (years)^a^	47.5 (23–73)	48.5 (18–77)	.698^§^
Gender (M:F)	165:27	45:9	.632
AFP (<20:≥20 μg/L)	60:132	6:48	.003*
HBsAg (positive: negative)	170:22	48:6	.943
HBeAg (positive: negative)	42:150	12:42	.957
Etiology (HBV: no-HBV)	173:19	49:5	0.889
PLT (10^9^/L) ^a^	142.5 (24–498)	158.0 (43–409)	.044^§^*
TBIL(umol/L) ^a^	13.6 (3.7–48.3)	14.4 (5.5–64.6)	.540^§^
ALB (g/L) ^a^	40.2 (24.7–50.6)	39.9 (28.6–50.3)	.824^§^
ALT (U/L) ^a^	49.8 (4.3–104.0)	49.6 (14.2–93.9)	.700^§^
Liver cirrhosis (yes: no)	126:66	34:20	.717
Tumor diameter (≤5:>5 cm)	69:123	12:42	.058
Tumor number(single: multiple)	143:49	23:31	<.001*
Encapsulation(complete: no)	45:147	3:51	.003^#^*
PVTT (yes: no)	20:172	5:49	.804
MVI (yes: no)	108:84	42:12	.004*
Differentiation (I-II: III-IV)	36:156	13:41	.387
BCLC staging (0/A:B:C)	135:37:20	22:27:5	<.001*
TNM staging (6th) (I:II:III)	69:77:46	6:19:29	<.001*

Note: *P<0.05 by χ^2^ test or Student *t* test, ^#^ Fisher’s exact test, ^§^Mann-Whitney test. ^a^ Median (range).

### Axl expression is correlated with poor prognosis following hepatectomy

The follow-up was ended on May 2011 (median, 42.3 months; range, 3.3–85.5 months). Up to the last follow up, 172 patients had tumor recurrence, 146 patients died and 5 were lost.

**Fig 3:**
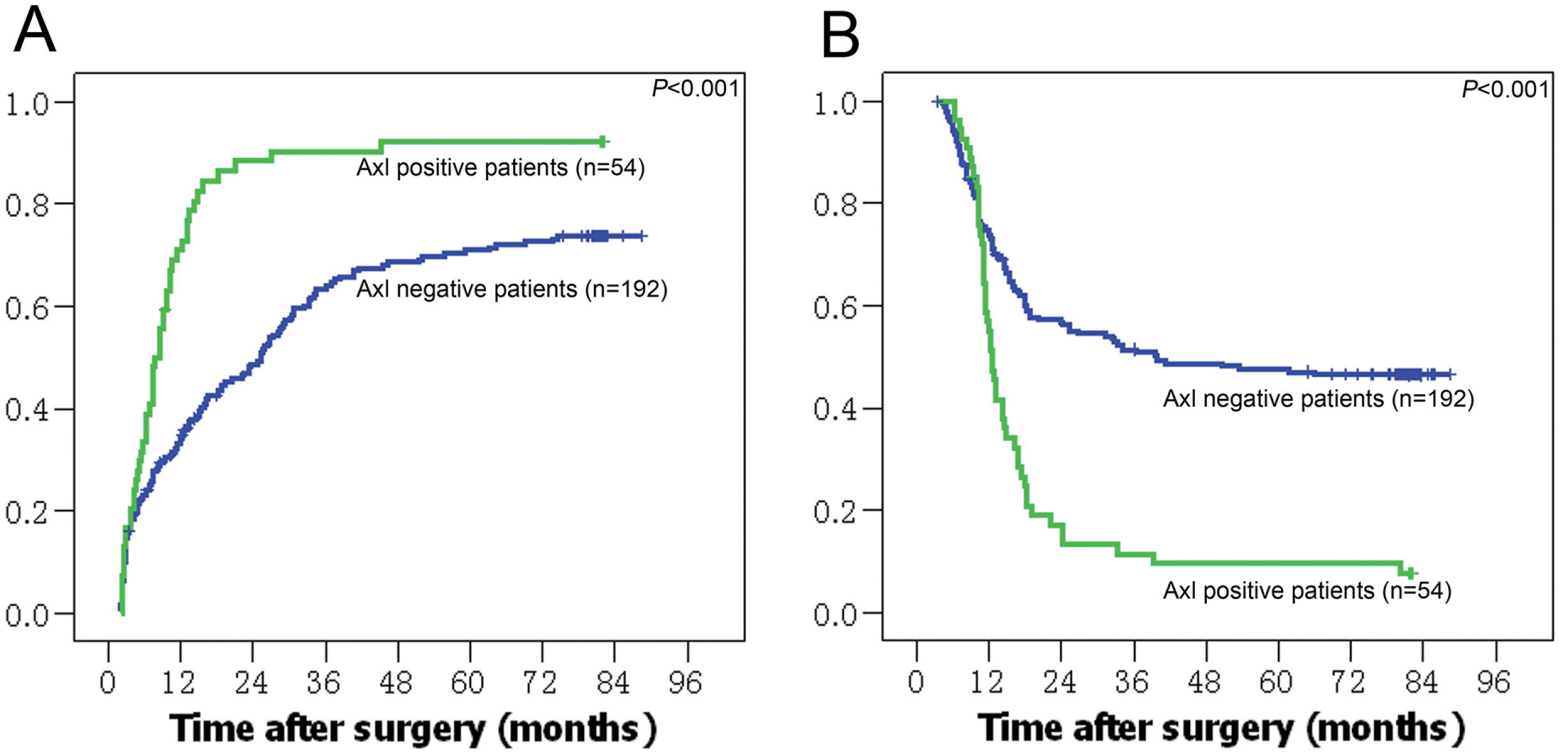
AXL expression and its association with prognosis in HCC patients. A, B: Kaplan-Meier curve of TTR and overall survival rates in patients with positive and negative AXL staining, respectively.

Patients with positive expression of Axl had higher recurrence rates (1-,3-,and 5-year recurrence rates: 73%, 90%, and 92% vs. 36%, 65%, and 71%, respectively, *P*<0.001), and lower overall survival rate (1-,3-,and 5-year survival rates: 45%,11%, and 9% vs. 70%,51%, and 48%, respectively, *P*<0.001) than those with negative expression (Fig 3A and 3B).

The result of univariate analysis was showed in Table 1. Multivariate analysis showed that the multiple tumors, absence of tumor capsule, PVTT, preoperative AFP positivity, and tumor diameter>5cm were the independent factors of postoperative tumor recurrence. The former three variables were also the independent factor for patients’ survival (Table 2). In addition, Axl expression was also an independent factor of both tumor recurrence (HR:1.725; 95% CI: 1.219–2.441) and patients^’^ survival (1.847; 1.291–2.642).

**Table 2:** Univariate and multivariate analyses for tumor recurrence and patient survival. **Abbreviations**: AFP, alpha-fetoprotein; PVTT, portal vein tumor thrumbus; MVI, microvascular invasion.

Variable	N	Tumor Recurrence				Overall Survival			
		Time ^†^	*P*^a^*	HR (95% CI)	*P*^b^*	Time ^†^	*P*^a^*	HR (95% CI)	*P*^b^*
AFP									
<20 μg/L	66	30.6±9.6	<.001	1.474 (1.029–2.111)	0.034	56.7±4.4	0.001		-
≥20 μg/L	180	11.8±1.8				15.9±1.3			
Tumor number									
single	166	25.3±3.1	<.001	1.480 (1.076–2.037)	0.016	49.8±2.8	<.001	3.230 (2.060–5.063)	<.001
multiple	80	7.8±0.6				12.2±0.5			
Tumor diameter									
≤5 cm	81	51.9±17.5	<.001	2.347 (1.636–3.368)	<.001	49.0±7.7	<.001	-	-
>5 cm	165	8.4±1.0				22.0±2.1			
Encapsulation									
presence	48	56.7±4.7	<.001	1.691 (1.079–2.650)	0.022	71.2±4.2	<.001	2.421 (1.343–4.365)	0.003
absence	198	12.2±1.5				15.3±1.3			
Differentiation									
I-II	49	-	-	-	-	52.7±5.3	0.029	-	-
III- IV	197	-				17.8±3.0			
MVI									
yes	150	10.3±1.9	<.001	-	-	13.7±1.1	<.001	-	-
no	96	32.4±6.1				58.3±3.6			
PVTT									
yes	25	3.2±0.2	<.001	4.971 (2.971–8.318)	<.001	7.4±0.9	<.001	4.605 (2.833–7.486)	<.001
no	221	20.4±3.2				32.4±7.0			
Axl expression									
positive	54	7.8±0.7	<.001	1.725 (1.219–2.441)	0.002	12.4±0.7	<.001	1.847 (1.291–2.642)	0.001
negative	192	25.3±3.2				49.9±2.7			

Notes:

^†^Results are presented as median ± standard error (months);

*Results are shown only for significant variables;

^a^, univariate analysis;

^b^, multivariate analysis.

There were 158 patients at early stage in the cohort according to the criteria of BCLC stage 0/A. Of them, 22 patients had positive expression and 135 patients had negative expression of Axl. The time to recurrence was 13.2±2.3 vs. 30.6±3.2 months (*P* = 0.01), and the survival time was 17.2±1.2 vs.57.1±3.1 months (*P* = 0.001) in the early stage HCC patients who had positive or negative Axl expression in the tumor, respectively.

## Discussion

In this study, we found that Axl was only expressed in the tumor tissues in a subset of patients with HCC. We also demonstrated *in vitro* that Axl expression in HCC cells tightly regulated tumor cell growth, migration and apoptosis. In the tissue array of 246 HCC patients undergoing hepatectomy, the patients with Axl expression showed more aggressive pathological features of tumor invasiveness and had a statistically higher tumor recurrence rate and lower survival rate at 5-year after surgery.

Axl has been reported as an important oncogene in human leukemia, prostate cancer, ductal carcinoma, and breast cancer et al [12,13,22–25]. However, its expression and biological function in HCC are less understood. Here, we found that over-expression of Axl would promote cell growth, likely due to its protecting tumor cells from cell apoptosis, based on an observation that the apoptotic rate was decreased in HCC cells with Axl over-expression in the study. Previous study has suggested that knock-down of Axl expression in pancreatic ductal adenocarcinoma cell lines increases radiation-induced cell apoptosis [22]. The PI3K/Akt pathway might be downstream of Axl activation, which might antagonizes apoptotic signals and promotes cell survival. It was reported that Akt phosphorylation level was greatly decreased when Axl signaling was inhibited in mesothelioma or metastatic breast cancer [23,24].

We also found that Axl expression could greatly affect HCC cell migration. It has been reported that Axl was selectively up-regulated in Hca-F cells, a mouse liver cancer cell line with high-metastatic potential to lymph nodes [16]. In breast cancer, Axl positively regulated epithelial-to-mesenchymal transition[13]. We also observed that over-expression of Axl induced HCC cell morphology changes into more invasive features and promoted cell migration through transwell. This finding is consistent with previous studies in breast cancer that Axl greatly regulates EMT, which might be through Slug and oncogeinc Ras signaling, because vimentin has been reported to govern Axl expression level to regulate these molecules [25–28]. During the preparation of this manuscript, two papers concerned the functions and mechanisms of Axl in HCC. Lee HJ et al found that Axl regulates tumor invasion through the transcriptional activation of Slug in HCC[29]. It was reported by Reichl P et al Axl knockdown severely impaired resistance to TGF-β mediated growth inhibition, cell invasion, and transendothelial migration in HCC[30]. However, whether Axl exerts its function through a different downstream signal pathway different from those in other cancer types remain to be investigated. Our unpublished paper also concentrated on this issue, which might be associated with angiogenesis, as shown in Table 1.

The above results about the role of Axl expression in HCC growth and invasiveness are further supported by the association between the expression of Axl and clinicopathological features. In the cohort of 246 patients, positive expression of Axl in their tumors was closely related with the absence of tumor capsule, presence of MVI, multiple nodules and high serum AFP concentration, which all pointed to an aggressively invasive property of the tumor (Table 1).

Reichl P et al found that soluble Axl revealed diagnostic value for very early stage hepatocellular carcinoma from multicenter analysis[31]. But the prognostic value of this molecular and whether the treatment choice could be made based on Axl expression or components related to Gas6/Axl signaling remained to be answered. Consistently, the highly invasive feature of tumors might explain why these patients with Axl expressing always had worse prognosis. Patients with positive expression of Axl had a significantly higher recurrence rate and lower overall survival rate than those with negative expression (5-year recurrence and survival rates: 92% vs 71%; 9% vs 48%; respectively, *P*<0.001 for both). Although there were only 54 patients (54/246, 21.9%) had Axl expression in their tumors, of those only one patient did not have tumor recurrence and 3 patients survived up to 3 years after hepatic resection. The specificity of this molecule in predicting HCC outcome was fully signified. Additionally, our results suggest that patients with Axl positive HCC may not be optimal candidates for partial hepatectomy. It was recommended that hepatectomy is indicated for early HCC patients such as those at BCLC 0/A stage [2,3]. Although the Axl expression had a positive relation with tumor stages, some patients at BCLC 0/A stage also had tumoral Axl expression accounting for about 40% of all positive patients. Both the time to recurrence and survival time are significantly shorter in the early stage HCC patients with positive Axl. Unlike advanced tumors, the prognostic predication of the early HCC remains a challenge for clinicians due to the similar clinicopathological features, Our finding suggests Axl expression might facilitate prognostic prediction for these patients. It is potentially feasible to evaluate the indication of hepatectomy for HCC according to the intratumoral Axl expression level, which can be achieved by biopsy sampling. Although this strategy somehow increases the risk of metastasis, it is a general pitfall for all intratumoral biomarker in clinical practices. From a more practical perspective, we can identify the patients at high risk of tumor recurrence and adapt more intensive monitoring procedure and investigate appropriate adjuvant therapies based on Axl expression test in resected tumor tissues.

There are limitations of this study: (1) the endogenous ligands that activates Axl in the intratumoral microenvironment are not discussed in this work. Additional investigations about Gas6 and protein S expression in HCC tissues will help answer this question; (2) although Axl was highly selective for prognosis prediction, it only expressed in a relatively small subset of HCC. Therefore, it would be important to combine Axl with other molecular or pathological parameters for comprehensive evaluation in clinical practices; (3) all clinical evaluation was based on data obtained from a single-institution in China and most of the enrolled patients had a background of HBV infection. It was reported that expression of Axl might be different based on the etiology of HCC[32]. In our study, most of the patients were HBV background, as shown in Table 1. But in our country, very small group of HCC patients were caused by obesity, HCV infection, or alcohol use. The interaction between Axl and HBV and whether this molecule has prognostic role in patients with HCV infection or other etiologies remain to be determined; (4) Although less addressing the mechanisms exerted by Axl, based on both the mechanism of Axl reported by few papers and the results of Table 1, we supposed that Axl was associated with angiogenesis in HBV related HCC. Some beneficial results have been published by the same research group[33].We are doing experiments on the relationship between Axl and HBV, and the in vivo environment such as a SCID mouse to study solid tumor formation (s.c. injection) and metastasis (tail vein injection) are also adopted. Some meaningful results were obtained, and a prospective clinical study was also registered based on Axl-associated molecular for its treatment guidance.

## Conclusions

Collectively, we for the first time demonstrated a close relation between intratumoral Axl expression and HCC invasiveness. Axl expression predicts a worse outcome of HCC patients undergoing partial hepatectomy.

## Supporting Information

S1 Text Supporting information of Materials and methods. (DOC)

S2 Text Supporting information of the construction of tissue microarray and evaluation of immunohistochemistry. (DOCX)

S1 Fig Densitometry analyses of 20 pairs of tumor and nontumorous tissue in western blot. (JPG)

S2 Fig Densitometry analyses of HCC cell lines in western blot. (JPG)

S3 Fig Cell growth curve of cells transfected with constructs as indicated by MTT assay. 1 stands for mock: control vector; 2 stands fo AXL-WT: the wild-type AXL; 3 stands fo si-Axl: transient transfection. All was *P*<0.05 versus mock,
(JPG)

S4 Fig Representative plots of flow cytometry and statistical results of cell apoptosis of cells transfected by constructs as indicated by propidium iodide and annexin V-FITC staining. The up down was the statistical results. A, Hep3B; B, HepG2; C, Hu7; D, L02. Bars, ±SD. *, *P*<0.05 versus mock, **, *P*<0.001 versus mock.
(JPG)

S5 Fig Statistical results of cells transfected with constructs as indicated in transwell assay. 1 stands for mock: control vector; 2 stands fo AXL-WT: the wild-type AXL; 3 stands fo si-Axl: transient transfection. All was *P*<0.05 versus mock.
(JPG)

S6 Fig Representative image of cells transfected with constructs as indicated in the wound healing assay. (JPG)

## Abbreviations

HCC: *hepatocellular carcinoma*
ASPH: *aspartyl-asparaginyl beta-hydroxylase*
Axl: *anexelekto*
Gas6: *growth arrest-specific gene 6*
EGF: *epidermal growth factor*
RTK: *receptor tyrosine kinase*
AFP: *α-fetoprotein*
TMA: *tissue microarray*
cDNA: *complementary DNA*
RT-PCR: *reverse transcriptase- polymerase chain reaction*
TNM: t*umor-node-metastasis*
BCLC: *Barcelona Clinic Liver Cancer*
TTR: *time to recurrence*
OS: *overall survival*
