# Supplementary material for: Axl Expression Stratifies Patients with Poor Prognosis after Hepatectomy for Hepatocellular Carcinoma
Source: PLoS One. 2016 May 16;11(5):e0154767. doi: 10.1371/journal.pone.0154767 (PMC4868325; doi:10.1371/journal.pone.0154767)
Supplement: S1 Text — (DOC) [file pone.0154767.s001.doc]

**Cell lines and expression constructs**

HCC cell lines, SMMC-7721, Hep3B, HepG2, and Hu7 were the kind gift of Dr.Lixin Wei of Tumor Immunology and Gene Therapy Center, the Eastern Hepatobiliary Surgery Hospital, Second Military Medical University. They were maintained at 37℃ in 5% CO2 in Dulbecco′s modified Eagle′s medium (DMEM) supplemented with 10% (v/v) FBS (Hyclone, Logan, UT), except SMMC-7721 which was maintained in RPMI 1640 with 10% FBS.

Wild type Axl (Axl-WT) was cloned into vector pcDNA3 (a kind gift of Dr. Arthur S. Polans, Department of Ophthalmology and Visual Science, Biomolecular Chemistry, University of Wisconsin Medical School, Madison, Wisconsin) and transfected into SMMC-7721 cells. Stable transfectants were established by selection with G418 with standard techniques. Monoclonal cell lines were selected according to the expression of Axl as monitored by immunoblot analysis. Small interfering RNA for Axl (Si-Axl, sense: GGA GAC CCG UUA UGG AGAA; antisense: UUC UCC AUA ACG GGU CUCC) designed and synthesized by Shanghai GeneChem Inc (Shanghai, China) was transient transfected into SMMC-7721. The interfered effect was confirmed by immunoblot analysis.

**RNA analysis**

Total RNA from each cell lines, 20 paired tumor tissue and nontumorous tissues were prepared by Trizol reagent (Invitrogen). Reverse transcription was performed on 1μg total RNA from each sample was used to synthesize first-strand cDNA using oligo(dT)_18_ primers and 200 units of SuperScript II (Life Technologies, Inc.) for extension. PCR amplification was performed with 1.25 units Ex Taq polymerase (TaKaRa, Dalian, China). The primers were as follows: Axl sense 5’GAG GAT GAA CAG GAT GAC TGG3’, antisense5’ ACG AAG GTC TGA TGT CCC AGA 3’. As an internal quantitative control of the gene expression, the β-actin:sense5’CAT CTC TTG CTC GAA GTC CA3’,antisense5’ATC ATG TTT GAG ACC TTC AAC A 3’ was used. All of the PCR products were resolved on a 1.8% agarose gel containing ethidium bromide.

Relative quantitation by real-time RT-PCR was performed using the SYBR-green detection of PCR products in real time with the Light Cycler (Roche Diagnostics, Meylan, France) according to the manufacturer’s instructions. The primers were the same as that used for the RT-PCR. The Axl and β-actin gene expressions of all cDNA samples were determined by fluorescence from SYBR green using the Light Cycler software Version 3.5 (Roche Diagnostics), and the ratios of Axl and β-actin gene expressions represented the normalized relative levels of Axl expressions.

**Western blot analysis**

Samples were lysed in T-PER Tissue Protein Extraction Reagent (Pierce, Rockford, IL) containing proteinase inhibitors (CalBiochem, San Diego, CA), and protein concentrations were measured with the BCA protein assay kit (Pierce). Total protein (20μg) were boiled for 5 min in SDS buffer, resolved by 8% SDS-PAGE, and transferred to nitrocellulose membranes. Membranes were blocked with 0.1M Tris (pH7.5), 0.9% NaCl, and 0.05% Tween-20 (TBST) containing 10% nonfat milk powder and then incubated with appropriate primary antibody (anti-Axl: R&D Systems, Inc; 1:500; β-actin: Santa Cruz Biotechnology；1:200), followed by incubation with anti-goat (rabbit) horseradish peroxidase-conjugated antibody (1:5000; Santa Cruz Biotechnology, CA). Finally, the probed proteins were detected using Western Blotting Luminol reagent (Santa Cruz Biotechnology).

**Analysis of cell growth in vitro**

The in vitro growth rate of SMMC-7721 cells expressing mock, Axl-WT, and Si-Axl was measured using the MTT assay in six replicates. Briefly, cells were seeded into 96-well plates at 2000 cells per well respectively. On the day of harvest, 100μl of spent medium was replaced with an equal volume of fresh medium containing 10% MTT 5 mg/ml stock (3-(4, 5-dimethylthiazol-2-y1)-2, 5-diphenyltetrazolium bromide; thiazolyl blue（Sigma, St. Louis, MO). Plates were incubated at 37°C for 4 h, then 100μl of DMSO (Sigma) was added to each well and plates shaken at room temperature for 10 min. The absorbance was measured at 570 nm.

**Apoptosis Assay**

SMMC-7721 cells (mock, Axl-WT, or Si-Axl) were washed, resuspended in the staining buffer, and examined with Vybrant Apoptosis Assay kit (Invitrogen) according to the manufacturer's instructions. Stained cells were detected by FACS (FACScalibur, Becton Dickinson, Mountain View, CA). The Annexin V-positive and PI-negative cells were regarded as apoptotic cells.

**Migration and invasion assay**

For boyden chamber invasion assay, SMMC7721 cells suspended in medium containing 0.1% FBS were plated on matrigel-coated 8-μm polypropylene filter inserts in the boyden chambers (Biocoat^TM^ Growth Factor Reduced Matrigel^TM^ Invasion Chamber, BD Biosciences, Bedford, MA). The bottom chamber contained RPMI 1640 with 10% FBS. After 24 h, the cells remaining in the insert were removed with a cotton swab, and the cells on the bottom of the filter were fixed and counted.

Cell monolayer wound healing assay was performed as described previously, with some modifications. In brief, confluent monolayers of cells were wounded with a uniform scratch. Cells were washed twice, and were permitted to migrate into the wound area for 24 hours. Wound closure was visualized with a microscope.
