# Supplementary material for: Axl Expression Stratifies Patients with Poor Prognosis after Hepatectomy for Hepatocellular Carcinoma
Source: PLoS One. 2016 May 16;11(5):e0154767. doi: 10.1371/journal.pone.0154767 (PMC4868325; doi:10.1371/journal.pone.0154767)
Supplement: S2 Text — (DOCX) [file pone.0154767.s002.docx]

**Tissue microarray and immunohistochemistry**

Tissue microarrays (TMA) were constructed as previously described[32]. The first antibody was purchased from the R&D Systems (1:50). Immunohistochemical staining was performed with the Dako Envision Plus System (Dako, Carpinteria, CA) according to the manufacturer’s instructions. Appropriate negative and positive controls were used. HCC was considered positive for Axl staining when >50% of tumor cells demonstrated ritten informed consent to par-highly condensed membranous and/or cytoplasmic immunoreaction deposits. A simple two-tier classification of “positive” and “negative” was used, as Axl has previously been described for TMA-based evaluation of immunohistochemical labeling.All sections were scored independently by two observers who were blind to the HCC clinico-pathological data. The concordance between scores from different sections of the same tumor was >90%.All discrepancies in scoring were reviewed, and a con-sensus was reached.
