# Supplementary figures and images for: Axl Expression Stratifies Patients with Poor Prognosis after Hepatectomy for Hepatocellular Carcinoma
Source: PLoS One. 2016 May 16;11(5):e0154767. doi: 10.1371/journal.pone.0154767 (PMC4868325; doi:10.1371/journal.pone.0154767)

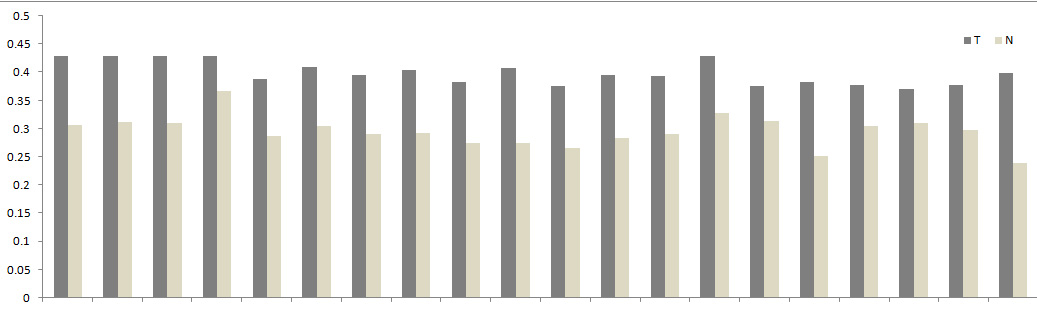

Supplement: S1 Fig — (JPG) [file pone.0154767.s003.jpg]

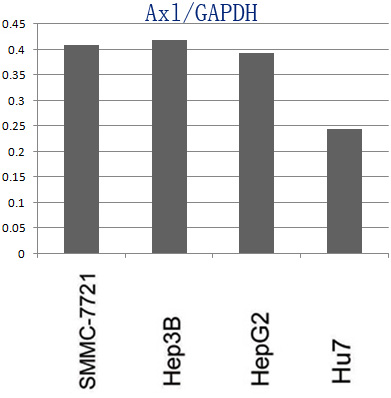

Supplement: S2 Fig — (JPG) [file pone.0154767.s004.jpg]

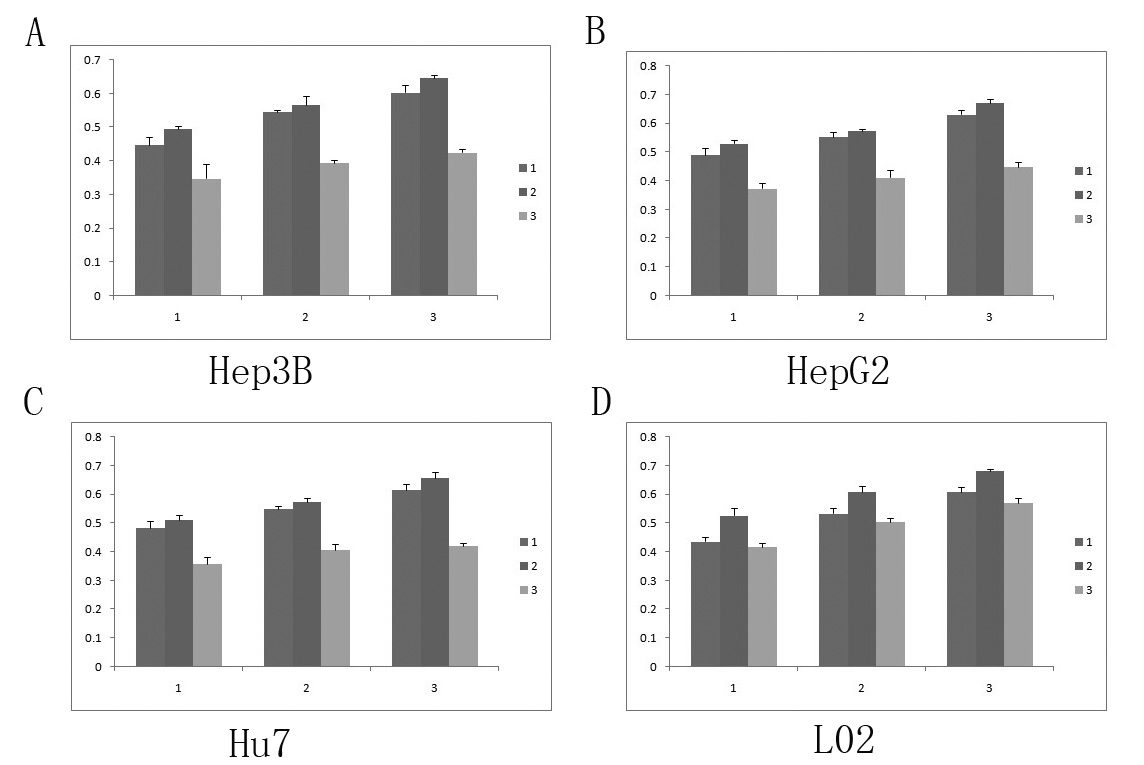

Supplement: S3 Fig — 1 stands for mock: control vector; 2 stands fo AXL-WT: the wild-type AXL; 3 stands fo si-Axl: transient transfection. All was P<0.05 versus mock, (JPG) [file pone.0154767.s005.jpg]

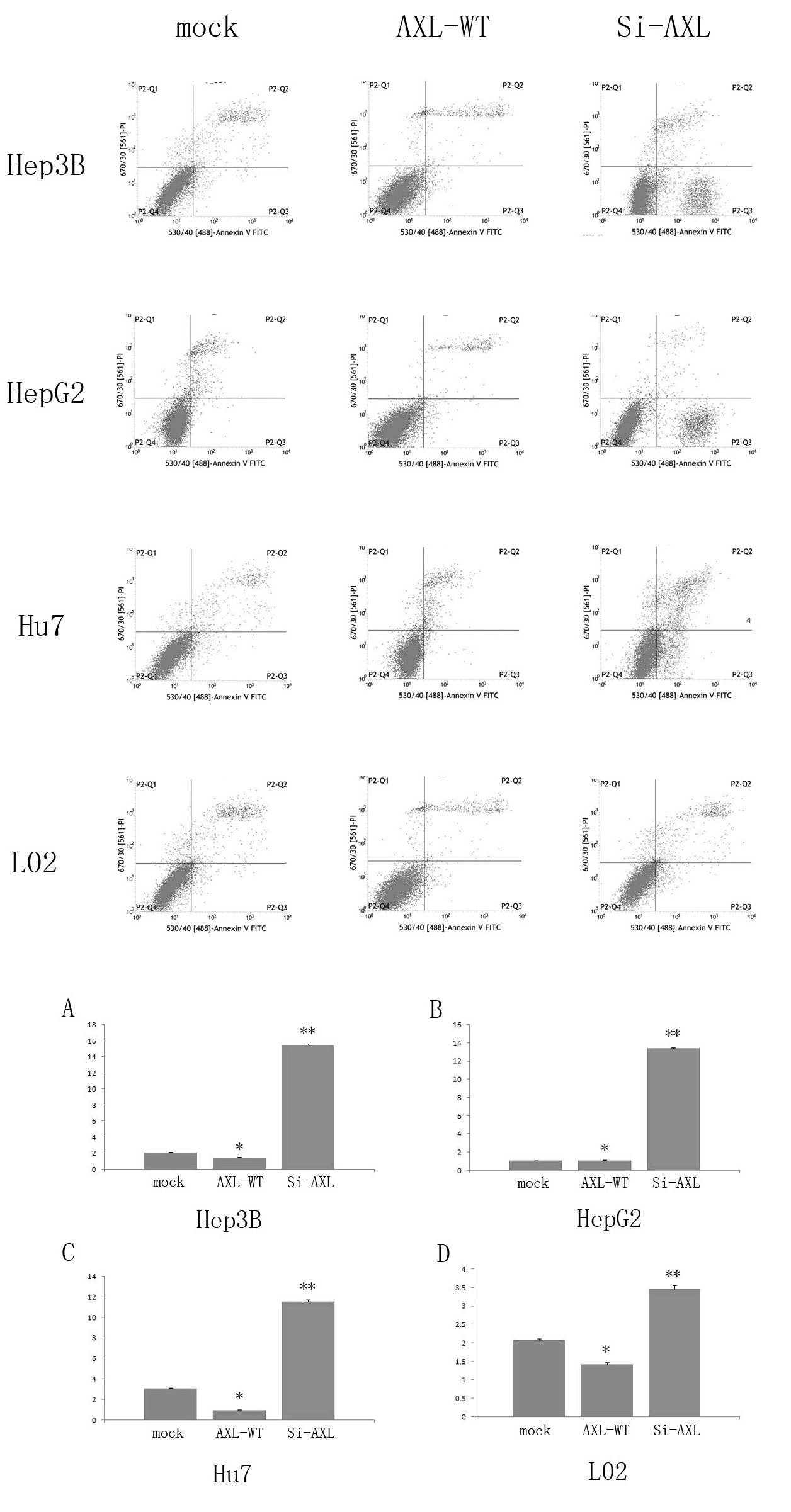

Supplement: S4 Fig — The up down was the statistical results. A, Hep3B; B, HepG2; C, Hu7; D, L02. Bars, ±SD. *, P<0.05 versus mock, **, P<0.001 versus mock. (JPG) [file pone.0154767.s006.jpg]

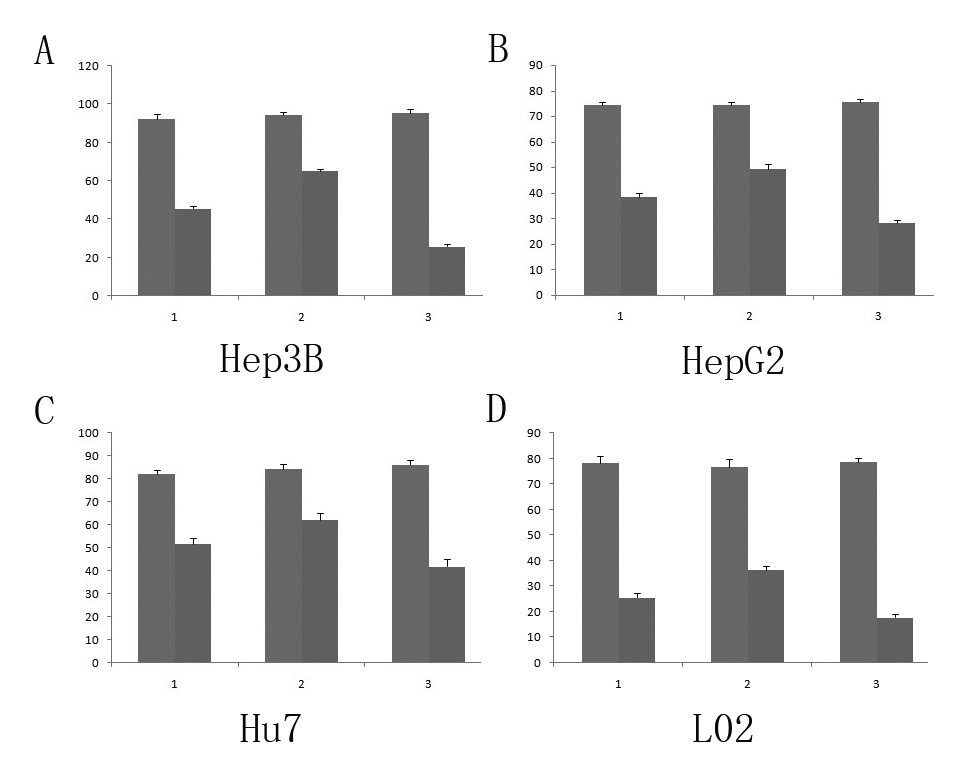

Supplement: S5 Fig — 1 stands for mock: control vector; 2 stands fo AXL-WT: the wild-type AXL; 3 stands fo si-Axl: transient transfection. All was P<0.05 versus mock. (JPG) [file pone.0154767.s007.jpg]

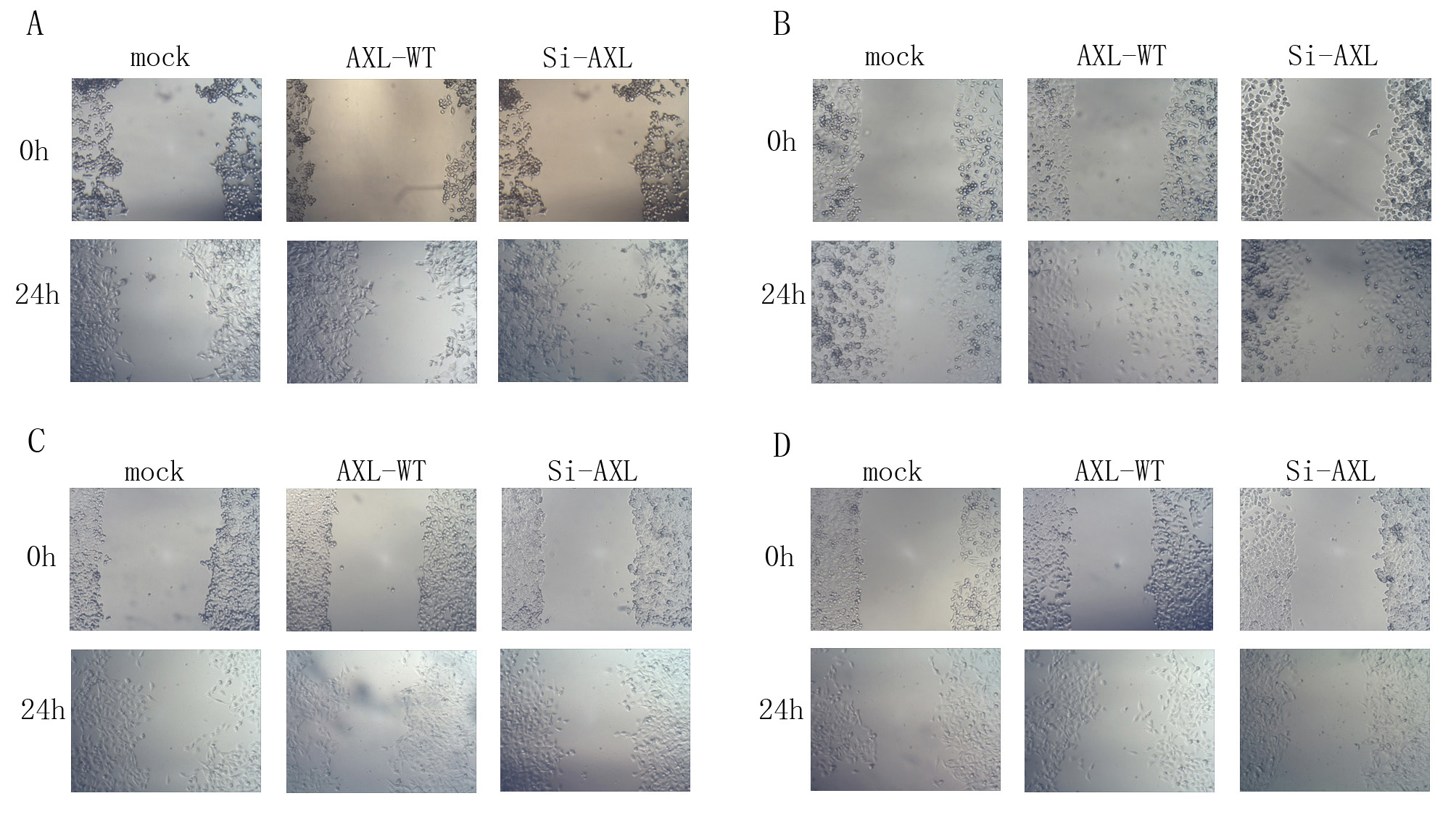

Supplement: S6 Fig — (JPG) [file pone.0154767.s008.jpg]
